# A multivariate plasma biomarker model for glioblastoma exclusion: a retrospective case-control study

**DOI:** 10.1016/j.tranon.2026.103079

**Published:** 2026-10-23

**Authors:** Jonathan Decarpentrie, Clara David, Nathalie Donis, Julien Favresse, Julien Cabo, Fabienne George, Matteo Riva, Lionel D’Hondt, Clotilde Brisbois, Jonathan Douxfils, NucleoGlio working group

**Affiliations:** a Clinical Pharmacology and Toxicology Research Unit, Namur Research Institute for Life Sciences, University of Namur, Namur, 5000, Belgium; b QUALIblood s.a., QUALIresearch, Liège, 4000, Belgium; c Department of Laboratory Medicine, Clinique St-Luc Bouge, Namur, 5000, Belgium; d Biobank, CHU UCL Namur Site Godinne, Université Catholique de Louvain, Yvoir, 5530, Belgium; e Department of Neurosurgery, CHU UCL Namur Site Godinne, Université Catholique de Louvain, Yvoir, 5530, Belgium; f Department of Oncology, CHU UCL Namur Site Godinne, Université Catholique de Louvain, Yvoir, 5530, Belgium; g Department of Biological Hematology, Centre Hospitalier Universitaire Clermont-Ferrand, Hôpital Estaing, Clermont-Ferrand, 63100, France

**Keywords:** Glioblastoma, GFAP, Neurofilament, Liquid biopsy, Biomarkers

## Abstract

**Background:**

Glioblastoma (GBM) remains the most aggressive primary brain tumour, with limited diagnostic tools able to capture its molecular heterogeneity in real time. Current clinical practice relies on MRI and invasive biopsies, which are poorly suited for early or dynamic disease monitoring. Sensitive and accessible blood-based biomarkers could offer a minimally invasive alternative to aid GBM diagnosis and exclusion.

**Methods:**

In this retrospective case-control study, plasma concentrations of seven neurological and inflammatory proteins (GFAP, UCH-L1, NfL, Tau, NSE, MPO, and PMN elastase) were quantified in 72 GBM patients and 102 healthy individuals (NAB-X biobank, n = 20; Clinique Saint-Luc Bouge, n = 82, age-matched) using ultrasensitive immunoassay platforms, complemented by conventional clinical laboratory assays and standard ELISA methods. Diagnostic accuracy was evaluated through ROC analysis and logistic regression modelling, with internal validation performed by 10-fold cross-validation and bootstrap resampling.

**Results:**

GFAP and NfL showed the highest individual discriminative performances (AUC = 0.995 and 0.957, respectively). A multivariate model integrating GFAP, NfL, Tau, NSE, MPO, and PMN elastase showed excellent discrimination between GBM and healthy controls (AUC = 0.999; sensitivity = 98.6%; specificity = 100%). The model’s negative predictive value remained above 0.999 in simulated population settings, supporting its use as a potential high-sensitivity exclusion tool.

**Conclusions:**

This study reports the possibility of using a plasma-based multivariate biomarker framework to discriminate GBM from healthy individuals with high sensitivity, providing proof-of-concept evidence supporting the future development of a plasma-based exclusion tool. This needs validation in clinically representative symptomatic populations. By integrating astrocytic, neuronal, and inflammatory markers into a single logistic model, this approach bridges analytical precision with clinical applicability, paving the way for a minimally invasive blood test to support diagnostic rule-out in neuro-oncology.

## Highlights

• Plasma GFAP and NfL show strong individual accuracy for GBM detection.
• A multivariate six-biomarker model achieves near-perfect GBM discrimination.
• The model shows high sensitivity for distinguishing GBM from healthy controls but requires validation in symptomatic non-GBM cohorts before clinical rule-out use.

## Importance of the study

Glioblastoma remains difficult to diagnose and monitor using current clinical tools, which rely primarily on MRI and invasive biopsies and often fail to capture tumour heterogeneity in real time. This study demonstrates that a combination of circulating astrocytic, neuronal, and inflammatory proteins can reliably distinguish glioblastoma patients from healthy individuals using a minimally invasive plasma sample in a retrospective case-control setting. By integrating six biomarkers into a single multivariate model, we observed an excellent discrimination and very high negative predictive values, supporting the use of this approach as proof-of-concept for a future exclusion tool following validation in symptomatic non-GBM populations. These findings expand upon prior literature focused on single biomarkers by showing that multimodal integration markedly improves diagnostic performance. The work highlights the translational potential of a blood-based test to support clinical triage, reduce diagnostic uncertainty, and complement imaging in early diagnostic pathways.

## Introduction

Glioblastoma (GBM), isocitrate dehydrogenase (IDH)-wildtype, CNS WHO grade 4, represents nearly 50% of all malignant brain tumours and remains the most aggressive primary brain malignancy, with a global incidence rate of around 4 per 100,000 person-years [1,2]. In Belgium, this incidence rate increases up to near 7 per 100,000 person-years [3]. The standard-of-care treatment, based on maximal safe resection followed by chemoradiotherapy (Stupp protocol), results in a median overall survival of only 15 months [3,4]. This bleak prognosis is largely attributed not only to the resistance and adaptability of the tumour but also to the limitations in current diagnostic and monitoring methods [5,6]. GBM is notoriously heterogeneous and undergoes substantial molecular evolution over time, especially under therapeutic pressure, necessitating dynamic disease monitoring [7]. However, current clinical practice relies on static tools, i.e., tissue biopsies and magnetic resonance imaging (MRI), that fail to capture tumour evolution in real-time and are limited in sensitivity and availability [8]. MRI, while standard, is often confounded by pseudoprogression, an inflammatory response mimicking tumour relapse, making radiological interpretation uncertain [9,10]. The clinical response in such cases frequently defaults to a "wait and see" strategy with repeated imaging or, in rare cases, advanced PET imaging, resources that are costly and not always available. Moreover, surgical biopsies are invasive and sometimes unfeasible, especially for tumours located in eloquent or deep brain regions. Even when feasible, stereotactic brain biopsy is associated with procedure-related morbidity, including hemorrhage and neurological deficits, which must be carefully weighed against the expected diagnostic benefit [11,12]. These clinical challenges underscore the urgent need for minimally invasive, dynamic, and accessible biomarkers capable of accurately diagnosing and monitoring GBM.

In this context, liquid biopsy has emerged as a transformative approach for tumour profiling through the analysis of tumour-derived components, such as circulating tumour DNA (ctDNA), cell-free DNA (cfDNA), microRNAs (miRNAs), and epigenetic markers, in biofluids [13,14]. cfDNA levels in serum have shown correlation with tumour burden and treatment response [15,16]. Several studies have identified specific miRNAs with diagnostic potential in GBM patients. miR-21, miR-128, and miR-342–3p were found to be significantly altered in the plasma of GBM patients compared to healthy controls, demonstrating high specificity and sensitivity in distinguishing glioma from normal samples [17]. Additionally, miR-15b has been implicated in GBM formation and progression [18]. Nonetheless, the application of blood-based ctDNA analysis in GBM remains severely constrained due to the blood-brain barrier (BBB), which limits the release of tumour DNA into the circulation. Plasma ctDNA levels in glioma patients are especially low (<20%), and current assays lack the sensitivity required for routine detection [19].

More specifically, in the context of GBM, cerebrospinal fluid (CSF) has emerged as the more reliable biofluid for ctDNA analysis in CNS tumours. Studies show that CSF-derived ctDNA captures intratumoral heterogeneity, clonal evolution, and resistance mutations with higher sensitivity (74–100%) and better concordance with tumour tissue [20,21]. However, CSF collection remains invasive, limiting its clinical utility for frequent monitoring. Considering these limitations, blood-based liquid biopsies emerge as the most practical and scalable sampling approach, providing a safer, simpler and a more readily repeatable option for both initial diagnosis and longitudinal monitoring [13].

Despite promising advances in molecular liquid biopsies strategies, there are still no clinically validated blood-based biomarkers for the reliable diagnosis or exclusion of GBM in clinical routine. Among all strategies, circulating protein biomarkers such as those associated with neuronal injury or neuroinflammation offer an interesting alternative. In our study, we hypothesize that a panel of neurological proteins could be detectable in plasma and discriminate GBM patients from healthy individuals. Several proteins reflecting neuroaxonal damage, glial activation, and inflammatory responses have shown diagnostic and prognostic value in neurological conditions, including traumatic brain injury and neurodegeneration. Notably, glial fibrillary acidic protein (GFAP), ubiquitin carboxy-terminal hydrolase L1 (UCH-L1), neurofilament light chain (NfL), Tau protein and neuron-specific enolase (NSE) are brain-specific proteins that can cross the blood-brain barrier upon injury or tumour-related disruption and have been proposed as promising blood-based biomarkers in central nervous system pathologies. NSE, a glycolytic enzyme predominantly expressed in neurons and neuroendocrine cells, has also been associated with traumatic brain injury, stroke, and brain tumours [[22], [23], [24], [25], [26], [27], [28], [29], [30], [31], [32], [33]]. Among additional protein candidates, neuron-specific enolase has also been investigated as a potential biomarker of neuronal injury and malignancy. In addition, inflammatory markers such as myeloperoxidase (MPO) and polymorphonuclear elastase (PMN elastase) may reflect complementary pathological processes relevant to GBM biology [25,26,[34], [35], [36]].

These protein biomarkers offer several advantages over nucleic acid–based approaches. Unlike ctDNA, which is often undetectable in plasma due to the restrictive nature of the blood-brain barrier and a shorten lifetime, proteins released from damaged or transformed neural cells are more stable and readily measurable using digital immunoassay platforms such as single molecular array platforms (Simoa) [37]. Moreover, the quantitative nature of protein assays facilitates the determination of clinically actionable thresholds, supporting real-time decision-making in clinical settings.

Importantly, the clinical objective of this work is not to establish a standalone diagnostic test for GBM, but to explore the feasibility of a high-sensitivity plasma-based exclusion strategy. Such an approach is intended to be used within a defined clinical pathway, in patients presenting with evocating neurological symptoms or undergoing MRI for suspected intracranial pathology, with the goal of safely deprioritizing aggressive brain tumours when biomarker levels fall below predefined exclusion thresholds.

Therefore, we assessed the diagnostic performance of seven candidate biomarkers, i.e. GFAP, UCH-L1, total Tau, NfL, NSE, MPO, and PMN elastase, in a retrospective case-control study of patients suffering from GBM and age-matched healthy individuals. Our aim was to evaluate their individual and combined performance for accurate, accessible, and real-time detection of GBM through blood-based testing.

## Material & methods

### Study design and participants

This retrospective study included a total of 174 peripheral blood samples obtained from four independent biobanks. Seventy-two samples from GBM patients were collected at the time of initial diagnosis, prior to any surgical intervention, radiotherapy, or systemic treatment. Among these, 61 samples were obtained from the CHU-UCL Namur biobank (Yvoir, Belgium), and 11 from Biomedica (Munich, Germany).

A total of 102 blood samples from healthy individuals were also analysed. These included 82 age-matched samples obtained from the clinical biology laboratory of Clinique Saint-Luc Bouge (Bouge, Belgium), and 20 samples provided by the NAB-X biobank (Namur, Belgium).

All blood samples were collected between 2010 and 2024 in K2-EDTA tubes and processed according to standardized operating procedures for plasma EDTA. Across all four biobanks, plasma samples underwent double centrifugation before storage and were aliquoted immediately after processing to minimise freeze–thaw cycles. All samples were analysed using identical analytical platforms and protocols. The study was conducted in accordance with the ethical principles of the Declaration of Helsinki and received central ethical approval from the CHU-UCL Namur Ethics Committee (approval number: 229/2023). For samples obtained from Clinique Saint-Luc Bouge, leftover clinical material was used for analytical validation and age-matched normal range determination, in accordance with the Belgian Law of 19 December 2008 on the procurement and use of human body material, which permits the secondary use of residual biological samples for research purposes without additional ethical approval under an opt-out framework. This study was not registered.

### Single molecular array (Simoa) assays

Glial fibrillary acidic protein, UCH-L1, NfL and Tau proteins were quantified in plasma using the Neurology 4-plex A assay (Quanterix®, Billerica, Massachusetts, USA), performed on the Simoa HD-X analyzer (Quanterix®) according to the manufacturer’s instruction.

Briefly, samples and calibrators were thawed on ice and vortexed before use. A 25 μL volume of each sample was loaded into a 96-well plate and incubated for 30 min at room temperature with paramagnetic beads conjugated to biomarker-specific antibodies under gentle agitation. After washing, biotinylated detection antibodies were added and incubated for another 30 min. Streptavidin-conjugated β-galactosidase was then introduced and incubated for 10 min, followed by final washes. Enzymatic signal was generated by the addition of a fluorogenic β-galactosidase substrate and measured using the Simoa HD-X platform.

### Neuron-specific enolase assay

Neuron-specific enolase (NSE) levels were measured in plasma samples using the Elecsys® NSE electrochemiluminescence immunoassay (Roche Diagnostics, Mannheim, Germany), performed on the cobas e analyser platform (Roche Diagnostics), in accordance with the manufacturer’s protocol.

Briefly, the assay is based on a sandwich principle using two monoclonal antibodies directed against NSE. Plasma samples were automatically incubated with a biotinylated anti-NSE antibody and a ruthenium-labelled anti-NSE antibody to form a sandwich complex. After the addition of streptavidin-coated microparticles, the immune complexes were magnetically captured and washed. Electrochemiluminescence was then induced by applying a voltage to the electrode, and the resulting signal was measured. The signal intensity is directly proportional to the NSE concentration in the sample.

### ELISA-based quantification of MPO and PMN elastase

Plasma concentrations of myeloperoxidase (MPO) were measured using the BMS2038INST Human MPO Instant ELISA kit (Thermo Fisher Scientific, Waltham, Massachusetts, USA), according to the manufacturer's instruction. Briefly, plasma samples were diluted 1:50. A 50 μL volume of the prediluted sample was added in the plate. After a 3-hour incubation at room temperature on a plate shaker, wells were washed four times. Next, 100 μL of 3,3′,5,5′-tetramethylbenzidine (TMB) substrate solution was added and incubated for 10 min in the dark. The enzymatic reaction was stopped, and absorbance was measured immediately at 450 nm using a microplate reader.

Plasma levels of PMN elastase were determined using the BMS269 Human PMN Elastase ELISA kit (Thermo Fisher Scientific, Waltham, Massachusetts, USA), following the manufacturer’s instructions. Samples were diluted 1:100. A 100 μL volume of each prediluted plasma sample was added to pre-coated microwells and incubated for 1 hour at room temperature on a plate shaker. Following four washing steps, 150 μL of horseradish peroxidase (HRP)-conjugated anti-α1-proteinase inhibitor (α1-PI) antibody was added and incubated for another hour. After additional washes, 200 μL of TMB substrate was added and incubated in the dark for 30 min. The reaction was stopped and absorbance was measured at 450 nm using a microplate reader.

### Statistical analysis: group comparisons

Biomarker concentrations from the Neurology 4-plex A, NSE, MPO and PMN ELISA kits were derived from raw signals through a 4- or 5-parameter logistic (4PL/5PL) curve fitting approach. Values below the lower limit of quantification (LLOQ) were replaced by the LLOQ/2 for downstream analyses. Results were log-transformed (after normality assessment using the D’Agostino-Pearson omnibus test) and presented as median ± standard deviation (SD). Group comparisons were assessed through Mann-Whitney U tests. A p-value of <0.05 was considered statistically significant. Data analyses were statistically evaluated using GraphPad Prism 10.3.0 (GraphPad Software, Massachusetts, USA).

### Statistical analysis: diagnostic performances and multivariate modelling

To determine whether combining circulating biomarkers could improve GBM discrimination, a multivariate logistic regression model was constructed using the glm() function (family = binomial) in R.

Pairwise correlations were visualized via corrplot, and Pearson’s correlation coefficients were reported. Predictor variables included GFAP, NfL, Tau, NSE, MPO, and PMN elastase. UCH-L1 was excluded from the final model due to its covariance with NfL and Tau (see *Results*).

Because circulating NfL is known to increase with age, an additional sensitivity analysis was performed to determine whether the association between NfL and GBM was independent of age. A logistic regression model including log-transformed NfL and age was fitted, and its diagnostic performance was compared with that of the unadjusted NfL model using receiver operating characteristic (ROC) analysis, likelihood-ratio testing, and Akaike's Information Criterion (AIC).

The final model was expressed as:

$$\mathrm{logit}\left(P_{\mathrm{GBM}}\right)=\beta_{0}+\beta_{1}\left(\mathrm{GFAP}\right)+\beta_{2}\left(\mathrm{NfL}\right)+\beta_{3}\left(\mathrm{Tau}\right)+\beta_{4}\left(\mathrm{NSE}\right)+\beta_{5}\left(\mathrm{MPO}\right)+\beta_{6}\left(\mathrm{PMN}\;\mathrm{Elastase}\right)$$

Model performance was quantified using ROC analysis, sensitivity, specificity, and diagnostic accuracy. Internal validation was achieved through two complementary resampling strategies implemented in the Classification And REgression Training (CARET) package:

– 10-fold cross-validation was applied to estimate mean AUC, sensitivity, and specificity across stratified data partitions, ensuring that each subset was used once for testing while the remaining samples were used for model training. This approach assesses the model’s generalizability and reduces overfitting bias.
– Non-parametric bootstrap resampling (1,000 iterations) was performed to evaluate the stability and dispersion of AUC values. By repeatedly drawing samples with replacement from the original dataset, this method quantifies the uncertainty of performance estimates and provides robust confidence intervals for the model’s discriminative ability.

Mean diagnostic metrics and 95% confidence intervals (CI) were obtained from the resampling distributions. Bootstrap-derived AUC distributions were visualized as Gaussian-like density plots to illustrate model robustness.

To further assess whether the observed model performance could arise from chance label structure, a permutation-based Y-scrambling analysis was performed (1000 iterations). The GBM/control labels were randomly permuted while preserving the biomarker matrix, and the complete modelling procedure was repeated for each permutation. The resulting AUC distribution was compared with that of the original model.

Model calibration was evaluated using complementary approaches. Calibration was first assessed graphically by bootstrap bias-corrected calibration curves generated with the rms package (1000 bootstrap resamples). Calibration intercept, calibration slope, Brier score and maximum calibration error (Emax) were reported to quantify model agreement between predicted and observed probabilities. The Hosmer-Lemeshow goodness-of-fit test was additionally performed as a descriptive assessment of calibration. Finally, the incremental value of the complete six-marker model over a reduced model including only GFAP and NfL was evaluated using the likelihood-ratio test and the AIC.

Cut-off values corresponding to a predicted GBM probability < 1% (p < 0.01) were calculated from the logistic regression equation. For each biomarker, the absolute concentration threshold was obtained by isolating the variable of interest while fixing all other predictors to their median control values.

All statistical analyses were performed using R version 4.3.1 (R Foundation for Statistical Computing, Vienna, Austria) through RStudio v2024.09.0. Receiver operating characteristic curves were generated using the pROC package, and AUCs were computed with 95% CIs derived from DeLong’s method. Significance was defined as p < 0.05 after two-tailed testing. All figures were generated in ggplot2 (v3.5.1) and gt (v0.10) for graphical summaries and tables. Scripts and anonymized datasets are available upon reasonable request to ensure full reproducibility. Requests must be addressed to the corresponding author.

### Reporting guidelines

This study was reported in accordance with the Transparent Reporting of a Multivariable Prediction Model for Individual Prognosis or Diagnosis (TRIPOD) statement and the Standards for Reporting Diagnostic Accuracy Studies (STARD) 2015 guidelines. Completed TRIPOD and STARD checklists are provided as **Supplementary material 1 & 2**.

## Results

### Population

The median age of GBM patients was 63.5 years (range 26 – 89), with a male-to-female ratio of 1:0.53. The control cohort had a median age of 54.5 years (range 18 – 85), and a male-to-female ratio of 1:1.27. Neither the age nor the sex has an impact on these biomarkers, except for NfL which showed a moderate correlation with age (**Supplementary material 3 & 4**). Tumours were predominantly located in the temporal (n = 18), parietal (n = 13), and frontal lobes (n = 11), with the remaining cases involving mixed or less frequent anatomical locations.

### Performance of individual biomarkers for glioblastoma detection

Plasma concentrations of all seven biomarkers were significantly elevated in GBM patients compared to controls (Fig. 1). GFAP showed the largest separation between groups, with median levels of 7667.0 pg/mL (Interquartile ranges – IQR: 1321.0–15,937.0) in GBM versus 81.5 pg/mL (IQR: 53.1–132.8) in controls (p < 0.0001). UCH-L1 and NfL were also markedly increased in GBM (UCH-L1: 261.3 pg/mL, IQR: 95.9–529.6 vs 28.5 pg/mL, IQR: 14.7–51.6; NfL: 73.1 pg/mL, IQR: 34.8–196.1 vs 7.9 pg/mL, IQR: 4.8–12.7; both p < 0.0001). Tau concentrations were moderately higher in GBM (4.0 pg/mL, IQR: 2.5–7.1) compared to controls (2.3 pg/mL, IQR: 1.6–3.1; p < 0.0001). NSE was significantly elevated (34.8 ng/mL, IQR: 10.9–63.9 vs 4.1 ng/mL, IQR: 2.6–8.3; p < 0.0001). Among the inflammatory markers, MPO showed an increase (32.5 ng/mL, IQR: 21.6–52.3 vs 20.7 ng/mL, IQR: 16.5–27.5; p < 0.0001), while PMN elastase exhibited a smaller but still significant difference (32.8 ng/mL, IQR: 23.9–48.9 vs 28.4 ng/mL, IQR: 22.8–34.4; p < 0.05).

**Fig. 1:**
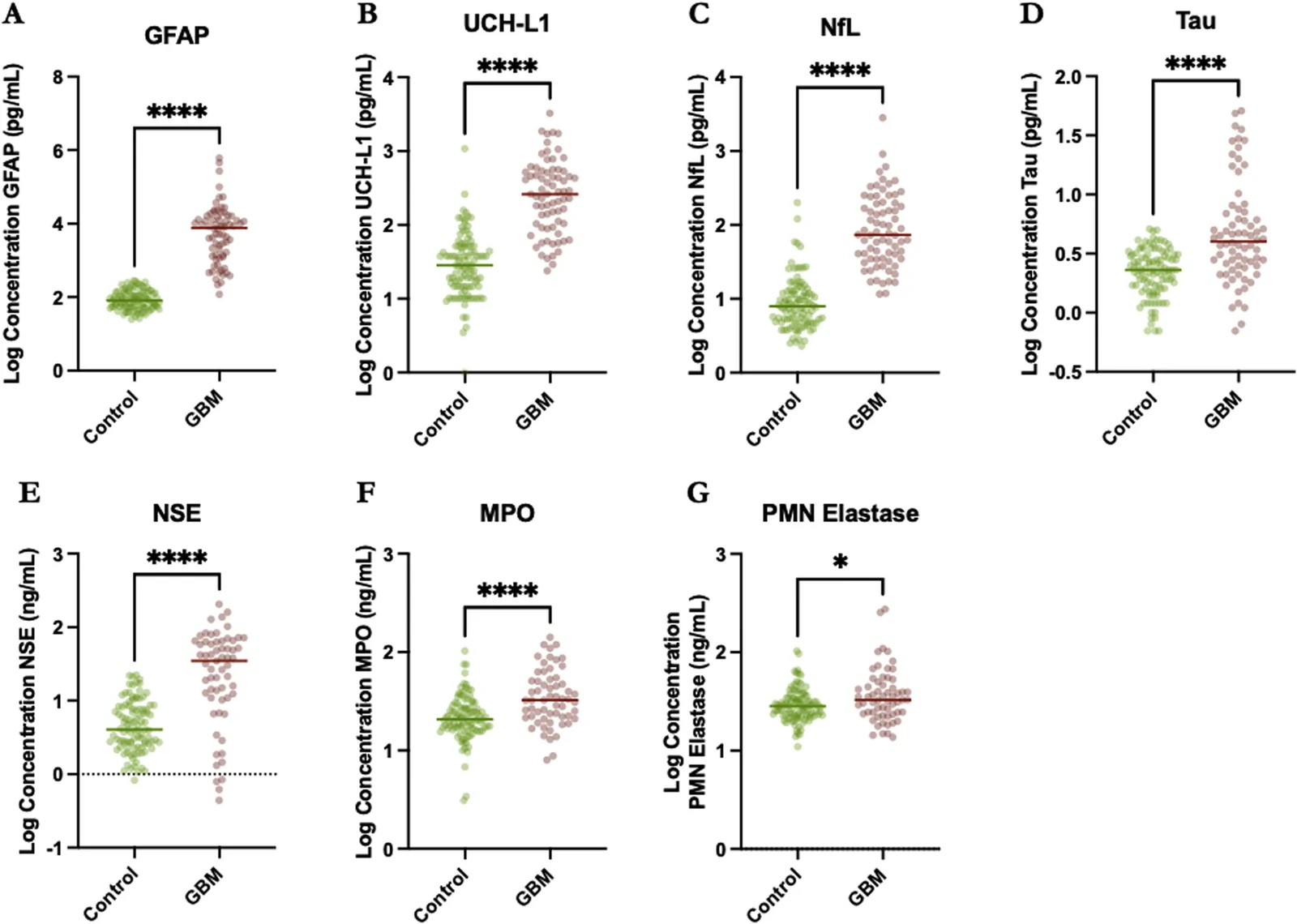
**Diagnostic performance of individual biomarkers for glioblastoma detection**. Log-transformed plasma concentrations of GFAP (**A**), UCH-L1 (**B**), NfL (**C**), Tau (**D**), NSE (**E**), MPO (**F**), and PMN Elastase (**G**) in control subjects (green) and GBM patients (red). Horizontal bars represent median values. Statistical significance was determined using the Mann-Whitney U test. **** p < 0.0001, * p < 0.05.

Given these marked differences in circulating concentrations, the diagnostic discriminative capacity of each biomarker was next evaluated. ROC curves were generated and the AUC computed (Fig. 2). Among the tested biomarkers, GFAP (AUC = 0.995 [95% CI: 0.987 – 1.000]), UCH-L1 (AUC = 0.925 [95% CI: 0.888 – 0.963]), and NfL (AUC = 0.957 [95% CI: 0.930 – 0.984]) displayed the highest diagnostic accuracy. Conversely, Tau (AUC = 0.757 [95% CI: 0.680 – 0.834]), NSE (AUC = 0.686 [95% CI: 0.598 – 0.795]), MPO (AUC = 0.694 [95% CI: 0.612 – 0.775]), and PMN elastase (AUC = 0.615 [95% CI: 0.527 – 0.704]) showed more modest discriminative performance. Comparative analysis of all ROC curves (Fig. 2) highlighted the superior classification performance of neuronal and astrocytic injury markers (GFAP, UCH-L1, NfL) compared to other markers.

**Fig. 2:**
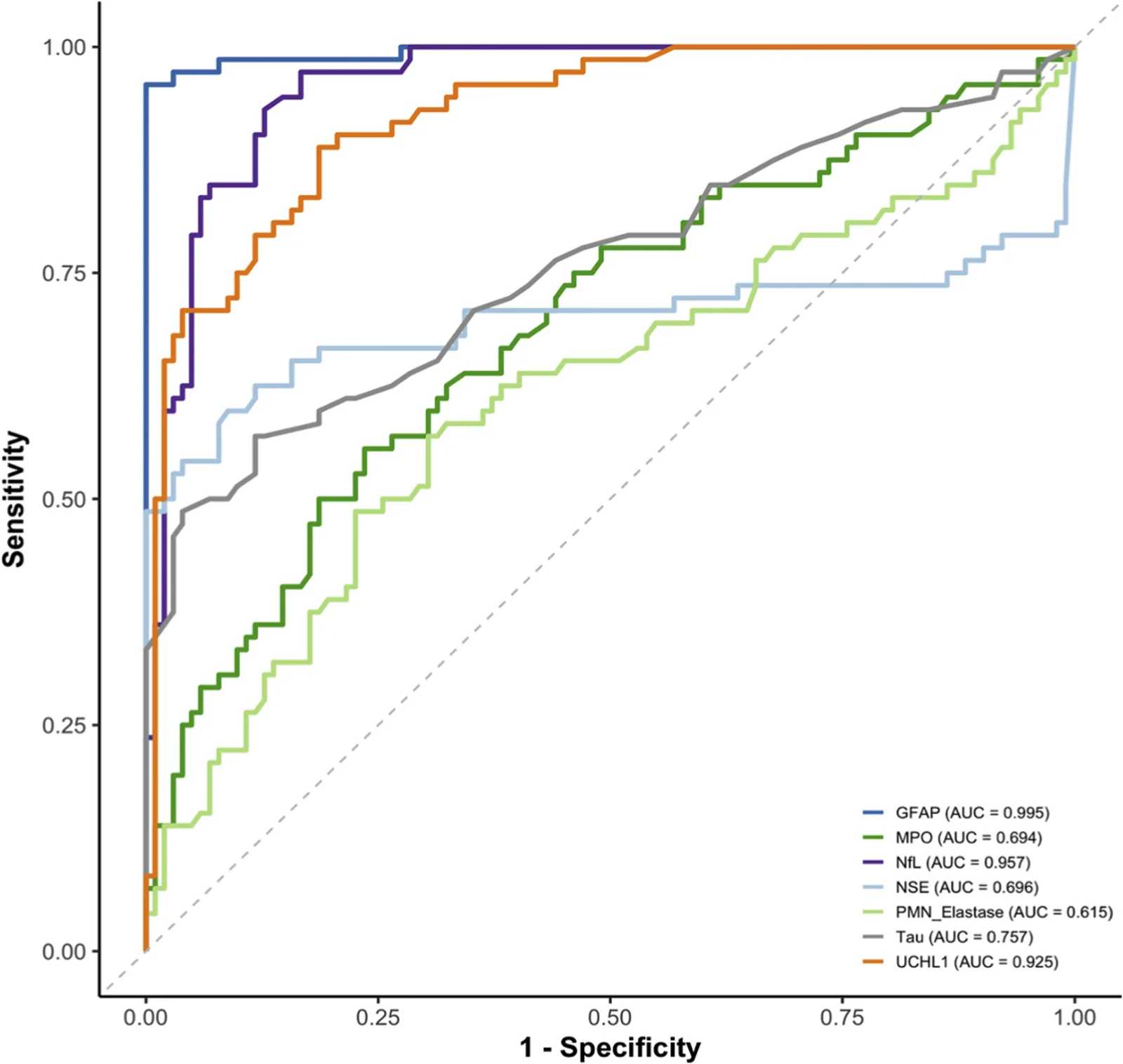
**Diagnostic performance of individual biomarkers for glioblastoma detection**. Receiver operating characteristic (ROC) curves comparing the discriminative performance of all evaluated biomarkers for distinguishing glioblastoma (GBM) from control samples. GFAP shows the highest diagnostic accuracy (AUC = 0.995), followed by NfL (AUC = 0.957) and UCHL1 (AUC = 0.925). In contrast, Tau (AUC = 0.757), NSE (AUC = 0.686), MPO (AUC = 0.694), and PMN elastase (AUC = 0.615) display lower discriminative power. The dashed diagonal line represents the line of no discrimination.

To further explore the potential clinical application of these markers for GBM exclusion, we determined cut-off values prioritizing maximal sensitivity (target Se = 1) (Table 1). Under this framework, GFAP and NfL thresholds of 119.85 and 11.55 pg/mL, respectively, achieved 100% sensitivity with specificities of 72.5% and 71.6%. MPO, Tau, and PMN elastase also reached near-maximal sensitivity (98.6–99.0%) but with markedly low specificity (≤ 3.9%). NSE demonstrated lower sensitivity (84.7%) at its exclusion cut-off (0.292 ng/mL).

**Table 1:** **Cut-off values for GBM exclusion based on maximal sensitivity.** Optimal cut-off concentrations for each biomarker were determined to prioritise maximal sensitivity (target Se = 1) for glioblastoma (GBM) exclusion. Values correspond to the concentration threshold yielding the indicated sensitivity and specificity.

Biomarkers	Cut-off value		Sensitivity	Specificity
***GFAP***	119.9	pg/mL	1.00	0.73
***NfL***	11.6	pg/mL	1.00	0.72
***UCH-L1***	23.0	pg/mL	1.00	0.43
***MPO***	7.4	ng/mL	0.99	0.04
***Tau***	0.8	pg/mL	0.99	0.03
***PMN Elastase***	12.3	ng/mL	0.99	0.01
***NSE***	0.3	ng/mL	0.85	0.01

Given the established age dependence of circulating NfL, an age-adjusted sensitivity analysis was performed. After adjustment for age, log-transformed NfL remained strongly associated with GBM (β = 6.36, p < 0.001), indicating that the observed increase in NfL was not solely explained by baseline age differences between groups. Inclusion of age resulted in a modest improvement in model fit (likelihood-ratio test: ΔDeviance = 4.34, p = 0.037; AIC: 99.46 vs. 101.80) and a slight increase in discriminative performance (AUC: 0.964 vs. 0.957).

### Multivariate modelling and validation of a plasma biomarker panel for glioblastoma exclusion

Following the demonstration of strong individual diagnostic performances, particularly for GFAP and NfL, the next analytical step focused on assessing whether multi-marker combinations could further enhance GBM detection accuracy. To ensure robustness of subsequent multivariate modelling, inter-marker dependencies were first examined. Pairwise correlation analysis (Fig. 3A) revealed marked associations among neuronal injury markers, notably UCH-L1, NfL, and Tau (r, Pearson’s correlation coefficient = 0.71 and r = 0.70, respectively), indicating a substantial degree of redundancy in their circulating profiles. In contrast, GFAP and the inflammatory mediators (MPO and PMN elastase) exhibited only weak to moderate correlations with other analytes (r < 0.45), suggesting more independent biological contributions. To mitigate multicollinearity and preserve model interpretability, UCH-L1 was excluded from subsequent combination analyses due to its high covariance with NfL and Tau.

**Fig. 3:**
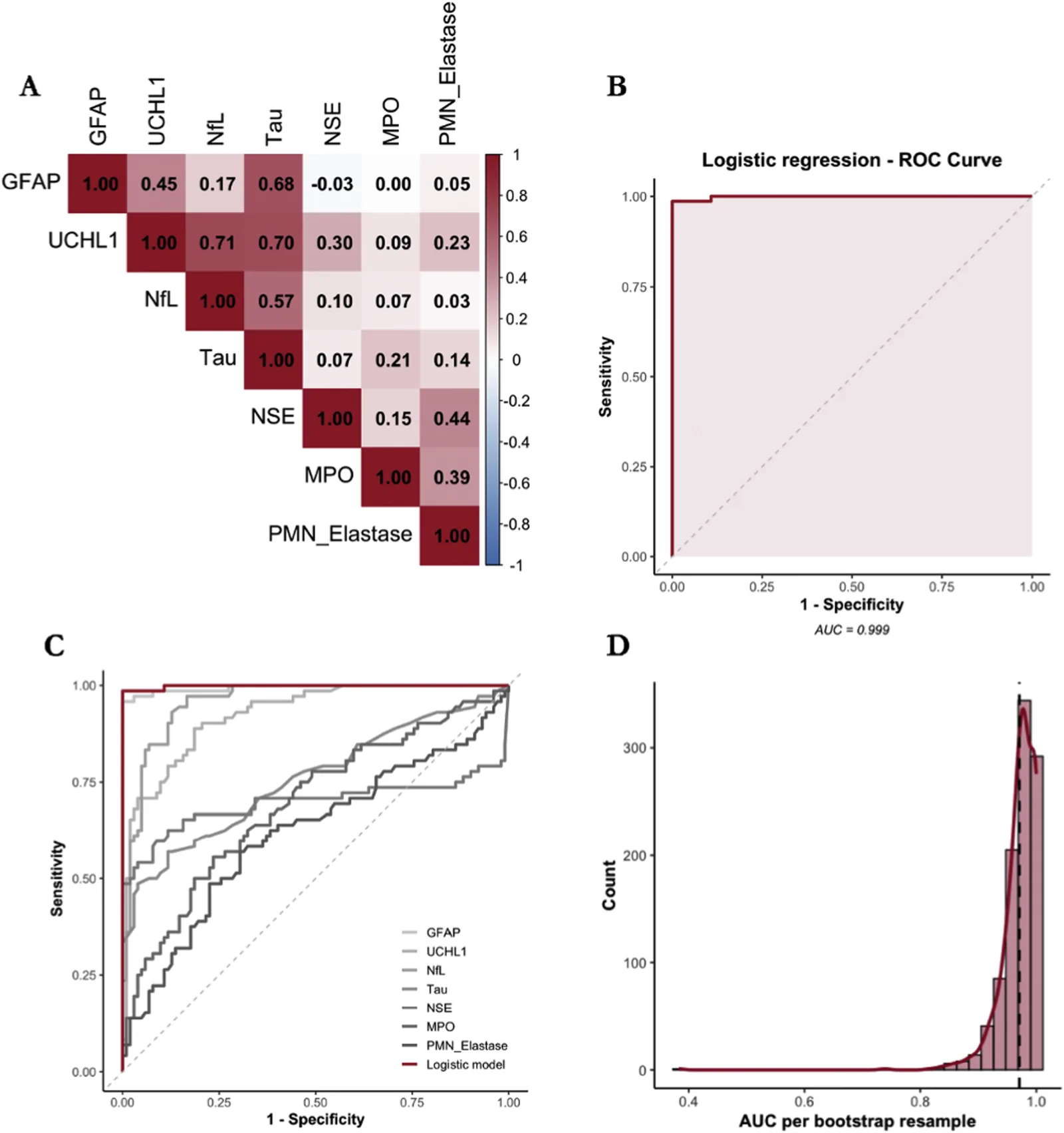
**Multivariate logistic regression model integrating circulating biomarkers for glioblastoma detection**. (A) Pairwise correlation matrix among all plasma biomarkers showing moderate to strong associations between neuronal injury markers (UCH-L1, NfL, Tau), while GFAP and inflammatory mediators (MPO, PMN elastase) displayed weaker intercorrelations. (B) Receiver operating characteristic (ROC) curve of the multivariate logistic regression model combining GFAP, NfL, Tau, NSE, MPO, and PMN elastase, demonstrating excellent discrimination between GBM and control samples (AUC = 0.999). (C) Comparative ROC curves of the individual biomarkers versus the multivariate model, highlighting the superior diagnostic performance of the combined signature. (D) Bootstrap distribution of AUC values across 1000 resamples, confirming the model’s stability and robustness (mean AUC = 0.971).

A multivariate logistic regression framework was constructed to interrogate whether integrating multiple circulating analytes could provide incremental diagnostic value over individual biomarker performance. The final logistic regression included six biomarkers – GFAP, NfL, Tau, NSE, MPO, and PMN elastase – representing distinct biological pathways involved in GBM pathophysiology.

The resulting multivariate model demonstrated a positive discrimination between GBM and control plasma profiles, with an AUC of 0.999 [95% CI: 0.995–1.000] (Fig. 3B-C). At the optimal probability threshold (Youden index), the classifier achieved a sensitivity of 98.6%, specificity of 100%. When transposed to a theoretical population with a GBM prevalence of 0.004%, the multivariate model maintained an almost perfect negative predictive value (NPV) of 0.999 [95% CI: 0.999–1.000] but a low positive predictive value (PPV) of 0.002 [95% CI: 0.000–0.003].

To assess model robustness and guard against potential overfitting, two complementary internal validation strategies were applied. First, 10-fold cross-validation yielded a consistently high predictive performance (mean AUC = 0.984 [95% CI: 0.924–1.000]; mean sensitivity = 0.970 [95% CI: 0.900–1.000]; mean specificity = 0.971 [95% CI: 0.857–1.000], with negligible inter-fold variance. Regarding predictive values, the cross-validation estimated a mean NPV of 0.999 [95% CI: 0.999–1.000] and mean PPV of 0.800 [95% CI: 0.001–1.000].

Second, non-parametric bootstrap resampling (n = 1000 iterations) provided an independent estimate of model stability, yielding a mean AUC of 0.971 [95% CI: 0.894–1.000], with average sensitivity and specificity of 0.959 [95% CI: 0.850–1.000] and 0.944 [95% CI: 0.793–1.000], respectively (Fig. 3D). Given a disease prevalence of 0.004%, resampling provided a mean NPV of 0.999 [95% CI: 0.999–1.000] and a mean PPV of 0.306 [95% CI: 0.001–1.000]. The resulting narrow bootstrap AUC distribution confirmed the internal reproducibility and resilience of the logistic model under repeated random resampling.

Permutation-based Y-scrambling further confirmed that the observed model performance was unlikely to result from random label assignment. Across 1000 label permutations, the mean AUC was 0.580 [95% CI: 0.474–0.662], whereas the original model achieved an AUC of 0.999. No permuted model reached the performance of the original classifier (empirical p < 0.001).

Model calibration demonstrated excellent agreement between predicted and observed probabilities. Bootstrap bias-corrected calibration showed minimal optimism, with a mean absolute calibration error of 0.015 (**Supplementary material 5**). The apparent calibration intercept and slope were −0.00 and 1.00, respectively, with a low Brier score (0.009) and a maximum calibration error (Emax) of 0.032. Consistent with these findings, the Hosmer-Lemeshow goodness-of-fit test showed no evidence of poor calibration (χ² = 0.019, p = 0.892).

To evaluate the contribution of the additional biomarkers beyond GFAP and NfL, the full six-marker model was formally compared with a reduced model including only GFAP and NfL. The full model demonstrated a significantly better fit according to the likelihood-ratio test (ΔDeviance = 10.11, df = 4, p = 0.039) and a lower AIC (27.68 vs. 29.79), supporting the inclusion of Tau, NSE, MPO and PMN elastase despite the modest gain in overall discrimination.

To translate the multivariate logistic model into a clinically interpretable framework, probabilistic cut-offs corresponding to a predicted probability of GBM below 1% (p < 0.01) were derived for each biomarker, assuming median values for the remaining covariates. These thresholds define quantitative exclusion boundaries at which the probability of disease becomes negligible, grounding clinical interpretation directly in the multivariate model rather than in univariate ROC analyses. As summarized in Table 2, the resulting cut-offs were 221.4 pg/mL for GFAP (β = 0.03), 380.8 pg/mL for NfL (β = 0.01), 10.5 pg/mL for Tau (β = 0.42), 15.8 ng/mL for NSE (β = 0.29), 65.8 ng/mL for MPO (β = 0.08), and 60.5 ng/mL for PMN elastase (β = –0.04). Application of the logistic model at the p < 0.01 exclusion threshold achieved complete separation between GBM and non-GBM cases (Table 3). All 72 GBM samples were correctly retained within the “non-excluded” category, while 79 of 102 control samples were appropriately classified as “excluded.” No GBM sample was erroneously assigned to the exclusion group, confirming that the model preserves maximal sensitivity while maintaining high discriminative power in control samples.

**Table 2:** **Cut-off values for GBM exclusion derived from the multivariate logistic regression model (p** **<** **1%)**. Cut-off values and corresponding β coefficients for each plasma biomarker included in the model. Thresholds were defined as the biomarker concentrations associated with a predicted probability of GBM below 1%, prioritizing maximal sensitivity for exclusion. Positive β values indicate higher biomarker levels increasing GBM probability, whereas negative β values denote inverse associations.

Biomarkers	Cut-off value		β coefficient
***GFAP***	221.39	pg/mL	0.03
***NfL***	380.78	pg/mL	0.01
***Tau***	10.52	pg/mL	0.42
***NSE***	15.76	ng/mL	0.29
***MPO***	65.79	ng/mL	0.08
***PMN Elastase***	60.47	ng/mL	−0.04

**Table 3:** **Classification performance of the logistic regression model (p** **<** **1%)**. Cross-tabulation of predicted versus actual diagnostic categories according to the logistic model using biomarker-specific exclusion cut-offs. All GBM cases were correctly identified (100% sensitivity), while 79 of 102 controls were excluded (specificity = 77.5%), supporting the model’s efficiency for GBM rule-out.

Real diagnostic	Included	Excluded
***GBM***	72	0
***Non-GBM***	23	79

To facilitate potential clinical translation and real-time interpretability, a web-based Shiny interface was developed. This interactive tool allows entry of individual plasma biomarker concentrations to compute the model-derived probability of GBM, display exclusion status according to probabilistic thresholds, and visualize the relative contribution of each marker to the logistic score. Such a framework provides an intuitive, clinician-friendly bridge between computational modelling and bedside applicability, supporting the potential deployment of plasma-based GBM risk estimation in translational and diagnostic settings.

## Discussion

The present study demonstrates that several circulating neurological and inflammatory proteins, particularly GFAP and NfL, are markedly elevated in patients with newly diagnosed GBM compared with healthy individuals, and that their integration into a multivariate logistic regression model enables excellent discrimination between GBM and healthy subjects within this retrospective case-control cohort. These results reinforce the concept that multiple circulating biomarkers reflecting distinct biological processes can cross a disrupted blood–brain barrier in GBM and be quantified using ultrasensitive immunoassays, offering a practical and minimally invasive avenue for disease assessment. The mechanisms underlying circulating GFAP elevation in GBM likely involve both tumour cell expression and reactive astrocytic responses, rather than a simple dependence on tumour size, necrosis, or overt blood–brain barrier breakdown. Previous studies have reported detectable plasma GFAP levels even in heterogeneous GBM presentations, supporting its relevance as a marker of diffuse astrocytic pathology. The absence of radiographic stratification in the present study limits mechanistic granularity and warrants dedicated investigation in future cohorts [30].

Because circulating NfL physiologically increases with age, we additionally evaluated its performance after adjustment for age [27,38]. Although age modestly improved model fit, NfL remained a highly significant independent predictor of GBM, indicating that its elevation reflects tumour-associated neuroaxonal injury beyond age-related baseline variation. These findings further support the robustness of NfL as a component of the proposed biomarker panel.

A key strength of this work lies in the demonstrated benefit of multimarker integration. Although GFAP alone demonstrated excellent discrimination, formal model comparison showed that the addition of the remaining biomarkers significantly improved overall model fit, as demonstrated by both likelihood-ratio testing and a lower AIC. Nevertheless, this incremental improvement was modest and should be interpreted cautiously until confirmed in additional validation cohorts.

From a clinical perspective, the relevance of the proposed biomarker model lies in its potential role as a high-sensitivity exclusion tool rather than as a diagnostic classifier. Accordingly, the present results should not be interpreted as demonstrating diagnostic performance in real clinical conditions, but rather as a proof-of-concept retrospective study. In routine practice, patients evaluated for suspected GBM typically present with progressive neurological symptoms and undergo brain MRI as the first-line investigation. However, MRI findings may be equivocal in early disease stages or overlap with non-neoplastic conditions, and access to advanced imaging may be limited in some settings. In this context, a plasma-based exclusion test could support early clinical triage by helping deprioritize the likelihood of an aggressive brain tumour when biomarker levels are below predefined exclusion thresholds, thereby complementing MRI rather than replacing it. Such an approach is intended to minimize false-negative results while supporting more efficient use of imaging resources during the initial assessment of symptomatic patients. Accordingly, the most relevant performance metrics for an exclusion strategy are sensitivity and negative predictive value (NPV). In the present dataset, to further evaluate the robustness and clinical relevance of our model, we deliberately varied the assumed prevalence from the general population rate of 0.004% up to 30%, reflecting settings ranging from screening-like conditions to clinically enriched cohorts. This exploration consistently showed reassuring NPV values (0.999 at 0.004%, 0.998 at 5%, 0.996 at 10%, and still 0.986 at 30%), while PPV increased as expected with prevalence (from 0.002 at 0.004% to 0.638 at 5% and 0.935 at 30%). These results highlight that even in scenarios where GBM prevalence is substantially higher, the model preserves very high rule-out performance, further supporting its intended clinical positioning (**Supplementary material 6**). This pattern – very high NPV with intrinsically low PPV – strongly supports the intended positioning of the model as a rule-out tool rather than as a confirmatory diagnostic test. Internal validation through cross-validation and bootstrap resampling further confirmed the stability of these characteristics, consistently showing high NPV and a wide range of PPV values, in line with the strong dependence of PPV on disease prevalence.

However, these predictive values must be interpreted cautiously in light of the study design. Because the control cohort consisted exclusively of healthy individuals, the estimated NPV and PPV derived from theoretical GBM prevalence may not fully reflect real-world performance in the intended clinical setting, where the test would be applied to symptomatic patients undergoing evaluation for suspected intracranial disease. In such clinically enriched populations, the pre-test probability of GBM is expected to be higher, which would increase PPV and modestly reduce NPV. Prospective validation in representative symptomatic non-GBM cohorts will therefore be required to establish clinically relevant diagnostic thresholds and predictive values.

Several important considerations must nevertheless be acknowledged when interpreting these findings. First, all GBM samples were collected at the time of initial diagnosis, prior to any therapeutic intervention, limiting confounding by surgery- or treatment-related tissue injury, without further clinical stratification (e.g., pre-surgical status, tumour burden, extent of symptoms, remission, or relapse). Because circulating biomarker levels likely vary across disease states, the absence of detailed staging information limits the capacity to generalize these findings to the full spectrum of GBM presentations, including postoperative follow-up and recurrence monitoring. Finally, while cross-validation, bootstrap resampling and bootstrap-based calibration analyses demonstrated excellent internal model stability and calibration, these procedures cannot substitute for independent external validation. The near-perfect discrimination observed in the present retrospective case-control cohort should therefore be interpreted cautiously until confirmed in prospective studies including clinically representative symptomatic populations.

Altogether, these findings support the feasibility of a plasma-based multivariate biomarker strategy for GBM exclusion. Although prospective external validation remains necessary before clinical implementation, the present results provide a strong proof-of-concept for further translational development.

## Conclusion

In summary, this proof-of-concept study provides a multivariate plasma biomarker model integrating the six-biomarkers model enabling discrimination of GBM patients from healthy controls with high sensitivity and negative predictive value when applied to population-level prevalence assumptions. These findings support this strategy as the basis for a potential high-sensitivity exclusion tool, pending validation in clinically representative populations. If confirmed in future studies, this multivariate approach could provide the basis for a minimally invasive blood test to assist clinicians in triaging symptomatic patients, optimizing imaging pathways, and reducing diagnostic uncertainty. However, translation into clinical practice will require validation in symptomatic, non-GBM populations and across distinct stages of disease, as well as rigorous assessment of pre-analytical factors and external calibration, including re-estimation of predictive values under clinically relevant prevalence conditions. In addition, given the major clinical challenge of distinguishing true tumour progression from treatment-related pseudoprogression, future studies should investigate whether longitudinal assessment of this plasma biomarker panel could aid in discriminating these entities during disease monitoring. With prospective external validation in clinically representative cohorts, this proof-of-concept approach may contribute to the future development of a more accessible and dynamic diagnostic strategy in neuro-oncology.

## Disclosure

J.Do reports personal fees and honorarium from Daiichi-Sankyo, Diagnostica Stago, DOASense, Gedeon Richter, Norgine, Portola, Roche, and Roche Diagnostics.

## CRediT authorship contribution statement

**Jonathan Decarpentrie:** Writing – review & editing, Writing – original draft, Validation, Project administration, Methodology, Investigation, Formal analysis, Conceptualization. **Clara David:** Investigation. **Nathalie Donis:** Project administration, Methodology. **Julien Favresse:** Investigation. **Julien Cabo:** Investigation. **Fabienne George:** Resources, Data curation. **Matteo Riva:** Resources. **Lionel D’Hondt:** Resources. **Clotilde Brisbois:** Investigation. **Jonathan Douxfils:** Writing – review & editing, Supervision, Project administration, Funding acquisition, Conceptualization.

## Declaration of competing interest

The authors declare the following financial interests/personal relationships which may be considered as potential competing interests:

Decarpentrie Jonathan reports financial support was provided by Walloon Region. Douxfils Jonathan reports a relationship with Daiichi Sankyo Inc that includes: consulting or advisory, paid expert testimony, and speaking and lecture fees. Douxfils Jonathan reports a relationship with Diagnostica Stago SAS that includes: consulting or advisory, paid expert testimony, and speaking and lecture fees. Douxfils Jonathan reports a relationship with DOASense that includes: consulting or advisory, paid expert testimony, and speaking and lecture fees. Douxfils Jonathan reports a relationship with Gedeon Richter Plc that includes: consulting or advisory, paid expert testimony, and speaking and lecture fees. Douxfils Jonathan reports a relationship with Norgine Ltd that includes: consulting or advisory, paid expert testimony, and speaking and lecture fees. Douxfils Jonathan reports a relationship with Portola Pharmaceuticals Inc that includes: consulting or advisory. Douxfils Jonathan reports a relationship with Roche that includes: consulting or advisory, paid expert testimony, and speaking and lecture fees. Decarpentrie Jonathan has patent pending to Qualiblood. Douxfils Jonathan has patent pending to Qualiblood. Favresse Julien has patent pending to Qualiblood. If there are other authors, they declare that they have no known competing financial interests or personal relationships that could have appeared to influence the work reported in this paper.

## Data Availability

All data generated for this study can be found within the manuscript. Scripts and raw data are available upon requests addressed to the corresponding author.
