# Supplementary material for: A multivariate plasma biomarker model for glioblastoma exclusion: a retrospective case-control study
Source: Transl Oncol. 2026 Oct 23;74:103079. doi: 10.1016/j.tranon.2026.103079 (PMC13720466; doi:10.1016/j.tranon.2026.103079)
Supplement: Supplementary file 1 [file mmc1.docx]

**SUPPLEMENTARY MATERIAL**

**Supplementary material 1 | TRIPOD+AI checklist**

**Supplementary material 2 | STARD checklist**

|  | **Section & Topic** | **No** | **Item** | **Reported on page #** |
| --- | --- | --- | --- | --- |
|  |  |  |  |  |
|  | **TITLE OR ABSTRACT** |  |  |  |
|  |  | **1** | Identification as a study of diagnostic accuracy using at least one measure of accuracy  (such as sensitivity, specificity, predictive values, or AUC) | 1-2 |
|  | **ABSTRACT** |  |  |  |
|  |  | **2** | Structured summary of study design, methods, results, and conclusions  (for specific guidance, see STARD for Abstracts) | 2 |
|  | **INTRODUCTION** |  |  |  |
|  |  | **3** | Scientific and clinical background, including the intended use and clinical role of the index test | 5-7 |
|  |  | **4** | Study objectives and hypotheses | 7 |
|  | **METHODS** |  |  |  |
|  | *Study design* | **5** | Whether data collection was planned before the index test and reference standard  were performed (prospective study) or after (retrospective study) | 7 |
|  | *Participants* | **6** | Eligibility criteria | 7-8 |
|  |  | **7** | On what basis potentially eligible participants were identified  (such as symptoms, results from previous tests, inclusion in registry) | 7-8 |
|  |  | **8** | Where and when potentially eligible participants were identified (setting, location and dates) | 7-8 |
|  |  | **9** | Whether participants formed a consecutive, random or convenience series | 7-8 |
|  | *Test methods* | **10a** | Index test, in sufficient detail to allow replication | 8-10 |
|  |  | **10b** | Reference standard, in sufficient detail to allow replication | 7-8 |
|  |  | **11** | Rationale for choosing the reference standard (if alternatives exist) | 7 |
|  |  | **12a** | Definition of and rationale for test positivity cut-offs or result categories  of the index test, distinguishing pre-specified from exploratory | 10-11, 15-16 |
|  |  | **12b** | Definition of and rationale for test positivity cut-offs or result categories  of the reference standard, distinguishing pre-specified from exploratory | 7-8 |
|  |  | **13a** | Whether clinical information and reference standard results were available  to the performers/readers of the index test | N/A |
|  |  | **13b** | Whether clinical information and index test results were available  to the assessors of the reference standard | N/A |
|  | *Analysis* | **14** | Methods for estimating or comparing measures of diagnostic accuracy | 10-12 |
|  |  | **15** | How indeterminate index test or reference standard results were handled | 10 |
|  |  | **16** | How missing data on the index test and reference standard were handled | 10 |
|  |  | **17** | Any analyses of variability in diagnostic accuracy, distinguishing pre-specified from exploratory | 14-16 |
|  |  | **18** | Intended sample size and how it was determined | 7-8 |
|  | **RESULTS** |  |  |  |
|  | *Participants* | **19** | Flow of participants, using a diagram | 12, no diagram |
|  |  | **20** | Baseline demographic and clinical characteristics of participants | 12-13 |
|  |  | **21a** | Distribution of severity of disease in those with the target condition | 12-13 |
|  |  | **21b** | Distribution of alternative diagnoses in those without the target condition | NA, healthy control |
|  |  | **22** | Time interval and any clinical interventions between index test and reference standard | 7-8 |
|  | *Test results* | **23** | Cross tabulation of the index test results (or their distribution)  by the results of the reference standard | 30-31 |
|  |  | **24** | Estimates of diagnostic accuracy and their precision (such as 95% confidence intervals) | 14-16, 30-31, 35 |
|  |  | **25** | Any adverse events from performing the index test or the reference standard | NA |
|  | **DISCUSSION** |  |  |  |
|  |  | **26** | Study limitations, including sources of potential bias, statistical uncertainty, and generalisability | 19 |
|  |  | **27** | Implications for practice, including the intended use and clinical role of the index test | 16-20 |
|  | **OTHER INFORMATION** |  |  |  |
|  |  | **28** | Registration number and name of registry | NA |
|  |  | **29** | Where the full study protocol can be accessed | NA |
|  |  | **30** | Sources of funding and other support; role of funders | 20-21 |
|  |  |  |  |  |

**Supplementary material 3 | Sex-related differences in plasma concentrations of biomarkers in healthy controls**

Box-and-whisker plots show circulating levels of GFAP, NfL, Tau, UCH-L1, MPO, PMN elastase, and NSE in men and women. Concentrations are displayed on a logarithmic scale to account for biomarker distribution. Median, interquartile range, and full data range are represented for each group. No statistically significant differences were observed between sexes for any biomarker (ns).

**Supplementary material 4 | Association between plasma biomarker concentrations and age in healthy controls**

Scatterplots display the relationship between age and circulating levels of GFAP, NSE, PMN elastase, Tau, NfL, MPO, and UCH-L1. Linear regression lines with 95% confidence intervals are shown for each biomarker. The coefficient of determination (R²) is indicated on each panel. Across biomarkers, age generally explained a limited proportion of variability, with the notable exception of NfL, which exhibited a moderate R² value.

**Supplementary material 5 | Bootstrap calibration curve of the six-marker logistic regression model**

Bootstrap bias-corrected calibration curve generated from 1000 bootstrap resamples demonstrating excellent agreement between predicted and observed probabilities. The apparent and optimism-corrected calibration curves remained close to the ideal line, indicating minimal overfitting. Mean absolute calibration error was 0.015, with an apparent calibration intercept of −0.00, calibration slope of 1.00, Brier score of 0.009 and Emax of 0.032.

| Projected prevalence | Negative predictive value | Positive predictive value |
| --- | --- | --- |
| 0.004 % | 0.999 [95% CI: 0.999–1.000] | 0.002 [95% CI: 0.000–0.003] |
| 0.100 % | 0.999 [95% CI: 0.999–1.000] | 0.032 [95% CI: 0.000–0.068] |
| 1.000 % | 0.999 [95% CI: 0.999–1.000] | 0.253 [95% CI: 0.040–0.465] |
| 5.000 % | 0.998 [95% CI: 0.996–1.000] | 0.638 [95% CI: 0.378–0.897] |
| 10.000 % | 0.996 [95% CI: 0.992–1.000] | 0.788 [95% CI: 0.600–0.976] |
| 20.000% | 0.992 [95% CI: 0.982–1.000] | 0.893 [95% CI: 0.786–1.000] |
| 30.000 % | 0.986 [95% CI: 0.969–1.000] | 0.935 [95% CI: 0.866–1.000] |

**Supplementary material 6 | Predicted negative and positive values across a range of projected disease prevalences**

The table reports the estimated negative predictive value (NPV) and positive predictive value (PPV) of the model at hypothetical prevalence levels ranging from 0.004% to 30%. Values are presented with corresponding 95% confidence intervals. As expected, NPV remains consistently high across all prevalence scenarios, whereas PPV increases proportionally with rising disease prevalence.
